# Multiparametrische Magnetresonanztomographie der Brust: Was bringt die Zukunft?

**DOI:** 10.1007/s00117-024-01390-1

**Published:** 2024-11-29

**Authors:** Daniela Prinz, Silvester J. Bartsch, Viktoria Ehret, Joachim Friske, Katja Pinker, Thomas H. Helbich

**Affiliations:** 1 Division of Molecular and Structural Preclinical Imaging, Department of Biomedical Imaging and Image-Guided Therapy, Medical University of Vienna, Währinger Gürtel 18–20, 1090 Wien, Österreich; 2 Division of Endocrinology and Metabolism, Department of Internal Medicine III, Medical University of Vienna, Wien, Österreich; 3 Division of Breast Imaging, Department of Radiology, Columbia University Vagelos College of Physicians and Surgeons, New York, USA

**Keywords:** Mammakarzinom, Brustkrebsdiagnostik, Multiparametrische Magnetresonanztomographie, Nichtinvasive Untersuchung, Krebsentstehung, Breast carcinoma, Breast cancer diagnostics, Multiparametric magnetic resonance imaging, Non-invasive examination, Cancer development

## Abstract

**Hintergrund:**

Die Kombination unterschiedlicher Methoden in der Magnetresonanztomographie (MRT) wird als multiparametrische MRT (mpMRT) beschrieben und nimmt in der Brustkrebsdiagnostik einen großen Stellenwert ein. Derzeit inkludiert die mpMRT die kontrastmittelverstärkte und diffusionsgewichtete MRT. Für eine umfassendere Charakterisierung der Schlüsselprozesse der Krebsentstehung sind zusätzliche MRT-Methoden, welche funktionelle Vorgänge auf zellulärer und molekularer Ebene erfassen, notwendig. Im Rahmen von präklinischen Studien werden MRT-Methoden, welche eine kontrastmittelfreie Evaluierung der Schlüsselprozesse auf metabolischer und molekularer Ebene ermöglichen, für zukünftige klinische Anwendungen entwickelt.

**Fragestellung:**

Wie sieht die mpMRT der Brust in Zukunft aus?

**Material und Methoden:**

Systematische Literaturanalyse fokussiert auf präklinische Forschung in Bezug auf mpMRT sowie Entwicklung und Modifizierung von nichtinvasiven MRT-Methoden.

**Ergebnisse:**

Einige vielversprechende MRT-Methoden für die Mammadiagnostik, die funktionelle als auch metabolische Fragen beantworten können, sind BOLD („blood oxygen level dependent“), IVIM („intravoxel incoherent motion“), DMI (Deuterium-MRT) und CEST („chemical exchange saturation transfer“). Eine Kombination, und somit ein multiparametrischer Ansatz, ermöglicht die nichtinvasive Differenzierung der Subtypen sowie die frühe Evaluierung des Therapieansprechens und ist somit entscheidend für den weiteren Verlauf der Krankheit.

**Schlussfolgerung:**

Standardisierung der Quantifizierung sowie Verbesserung und Erweiterung der MRT-Methoden ermöglichen solch eine multiparametrische, funktionelle und metabolische Einschätzung des Tumors. Viele davon werden zunächst in der Präklinik entwickelt, bevor die Translation in die Klinik erfolgen kann.

## Abstract (en)

**Background:**

The combination of different MRI methods is described as multiparametric MRI (mpMRI) and plays a significant role in breast cancer diagnostics. Currently, mpMRI includes contrast-enhanced and diffusion-weighted MRI. For a more comprehensive characterization of the key processes involved in cancer development, additional MRI methods that capture functional processes at the cellular and molecular levels are necessary. In the context of preclinical studies, MRI methods that enable contrast-free evaluation of key processes at the metabolic and molecular levels are being developed for future clinical applications.

**Objectives:**

What does multiparametric MRI in breast cancer look like in the future?

**Methods:**

Systematic literature analysis focusing on preclinical research with regard to mpMRI as well as development and modification of noninvasive MRI methods.

**Results:**

Some of the most promising MRI methods for the evaluation of breast cancer that can answer functional and metabolic questions are BOLD (blood oxygen level dependent), IVIM (intravoxel incoherent motion), DMI (deuterium metabolic imaging) and CEST (chemical exchange saturation transfer). A combination and, therefore, a multiparametric approach allows for a noninvasive differentiation of breast cancer subtypes and early detection of treatment response which is crucial for the future development of the disease.

**Conclusion:**

Standardization of quantification methods as well as improvement and expansion of MRI methods enable such a multiparametric, functional, and metabolic evaluation of the tumor. Many of these are initially developed in preclinical settings before they can be translated into clinical practice.

Die Kombination mehrerer Methoden der Magnetresonanztomographie wird als multiparametrische MRT (mpMRT) bezeichnet und nimmt in der Brustkrebsdiagnostik einen großen Stellenwert ein. Derzeit umfasst die mpMRT zumeist kontrastmittelverstärkte und diffusionsgewichtete Verfahren [1, 2]. Für eine umfassendere Charakterisierung der Schlüsselprozesse der Krebsentstehung [3] und wiederholte Erfassung der intratumoralen Heterogenität während des Therapieverlaufs sind zusätzliche MRT-Methoden, welche funktionelle Vorgänge auf zellulärer und molekularer Ebene erfassen, notwendig. Im Rahmen von präklinischen Studien werden diese für zukünftige klinische Anwendungen entwickelt. Einige dieser vielversprechenden MRT-Methoden werden in diesem Artikel beschrieben.

## Hintergrund

In der aktuellen Brustkrebsdiagnostik wird die Nadelbiopsie des Tumorgewebes als Goldstandard angesehen, wobei eine ganzheitliche Erfassung insbesondere der Brustkrebsheterogenität nicht möglich ist. Die nichtinvasive bildgebende Charakterisierung verschiedener Schlüsselprozesse der Krebsentstehung, wie Hypoxie, Neoangiogenese und Stoffwechseländerungen, z. B. des Glukosestoffwechsels, würde eine wichtige Ergänzung zur invasiven Brustdiagnostik bieten. Schon jetzt erlaubt die mpMRT neben der morphologischen Bildgebung die Erfassung von funktionellen Vorgängen auf zellulärer und molekularer Ebene. Derzeit umfasst die klinische mpMRT der Brust primär die kontrastmittelverstärkte und diffusionsgewichtete MRT [1–3], und das Potenzial der mpMRT ist noch nicht vollständig ausgeschöpft.

MRT-Techniken, welche eine kontrastmittelfreie Evaluierung der Schlüsselprozesse der Krebsentstehung auf metabolischer und molekularer Ebene ermöglichen, werden derzeit in der Präklinik entwickelt, modifiziert und in Hinblick auf ihre Reproduzierbarkeit optimiert [4]. Die präklinische Forschung und eine erfolgreiche Translation der damit gewonnenen Erkenntnisse bilden somit die Grundlage neuer mpMRT-Protokolle und einer verbesserten klinischen Diagnostik.

## Hyperoxische BOLD-MRT

Ein Merkmal besonders aggressiver Tumore ist die Entwicklung hypoxischer Areale. Durch das schnelle Wachstum des Tumors kommt es zu immaturen, porösen Blutgefäßen und somit zu einem chronischen Sauerstoffmangel in Tumorarealen, der mit Therapieresistenz und einer schlechten Prognose einhergeht. BOLD(„blood oxygen level dependent“)-MRT ist eine Methode, die sich den beschleunigten Abfall des T_2_*-Signals aufgrund der paramagnetischen Eigenschaft von Desoxyhämoglobin zunutze macht [5]. Um das Ausmaß des Sauerstofftransports durch die Blutgefäße in das Tumorgewebe zu charakterisieren, wird in einem zweistufigen Messverfahren die Relaxationsrate R_2_* = 1/T_2_* des Tumors gemessen. Diese wird vor und nach Einatmen eines hyperoxischen Gases über eine Atemmaske [6], meist 100 % Sauerstoff oder Carbogen (95 % O_2_ und 5 % CO_2_), quantifiziert. Die Differenz der beiden Signale ist größer, wenn stark vaskularisierte Tumorareale eine höhere Konzentration Oxyhämoglobin als hypoxische Areale infolge der Einatmung hyperoxischen Gases aufweisen [7].

### Anwendung bei Brustkrebs

**Abb. 1:**
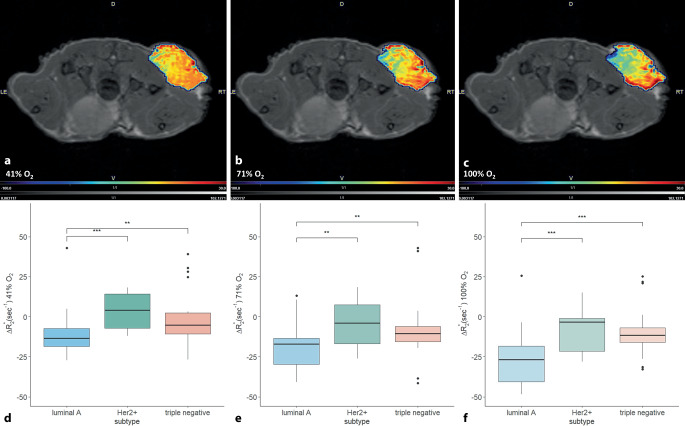
Auf axiale *T*_*2*_-gewichtete anatomische Referenzbilder überlagerte BOLD-MRT-Bilder eines triple-negativen Brustkrebs-Xenografttumors im Mausmodell (**a**) *Δ*R_2_* Parameterkarte bei 41 % Sauerstoff, (**b**) *Δ*R_2_* Parameterkarte bei 71 % Sauerstoff und (**c**) *Δ*R_2_* Parameterkarte bei 100 % Sauerstoff. (**d**–**f**) Boxplots zeigen die Unterschiede in der Änderung von R_2_* zwischen Brustkrebssubtypen nach der Gabe hyperoxischen Gases bei (**d**) 41 % O_2_, (**e**) 71 % O_2,_ (**f**) 100 % O_2_. (Mod. nach [10], **p < 0,01; ***p < 0,001)

Die ersten klinischen Studien zeigten bereits das Potenzial von BOLD-basierten Methoden in der Brustkrebsdiagnostik auf [8, 9]. Bei präklinischen Brustkrebsmodellen konnten Bartsch et al. [10] zeigen, dass mittels der hyperoxischen BOLD-MRT eine signifikante Unterscheidung zwischen Luminal A, HER2+ und triple-negativen Brustkrebstypen möglich ist. (Abb. 1; [10, 11]). Die BOLD-Messungen wurden nach Inhalation von unterschiedlichen Sauerstoffkonzentrationen durchgeführt, wobei die Unterscheidung der Brustkrebssubtypen bereits ab 41 % O_2_ gelang (Abb. 1d). Die Modulation der Atemgase kann technisch wie auch physisch bzw. psychisch eine Herausforderung sein. Kauczor [12] konnte nach der Inhalation von hyperpolarisiertem Helium in der MRT zeigen, dass die Darstellung der Lunge und pathologischer Prozesse möglich ist. Dabei erwies sich Helium als nebenwirkungsfrei und leicht anwendbar [12]. Rakow-Penner et al. [13] konnten zeigen, dass die Einatmung von reinem Sauerstoff von Patientinnen gut toleriert wird.

Eine niedrig dosierte Inhalation von reinem Sauerstoff (ca. 40 % O_2_) wie von Bartsch et al. [10] beschrieben, könnte sich somit als nichtinvasive und kontrastmittelfreie hyperoxische BOLD-MRT für eine Translation in die Klinik eignen und ließe sich in das Spektrum der mpMRT leicht integrieren.

## Von DWI zu IVIM-MRT

### Diffusionsgewichtete Bildgebung (DWI)

Die DWI basiert auf dem Einfluss ungerichteter Bewegung (Brownsche Molekularbewegung) der Wassermoleküle im Gewebe auf das MRT-Signal und hat bereits ein breites Anwendungsspektrum in der MRT-Diagnostik. Das gemessene Signal hängt dabei vom Diffusionskoeffizienten (D) des Gewebes und technischen Parametern der Pulssequenz ab, die im b‑Wert zusammengefasst werden. Ein ADC („apparent diffusion coefficient“) kann bereits mit zwei b‑Werten berechnet werden. In der Brustkrebsbildgebung haben sich die Werte 0 und 800 s/mm^2^ bewährt [14].

### „Intravoxel incoherent motion“ (IVIM)

**Abb. 2:**
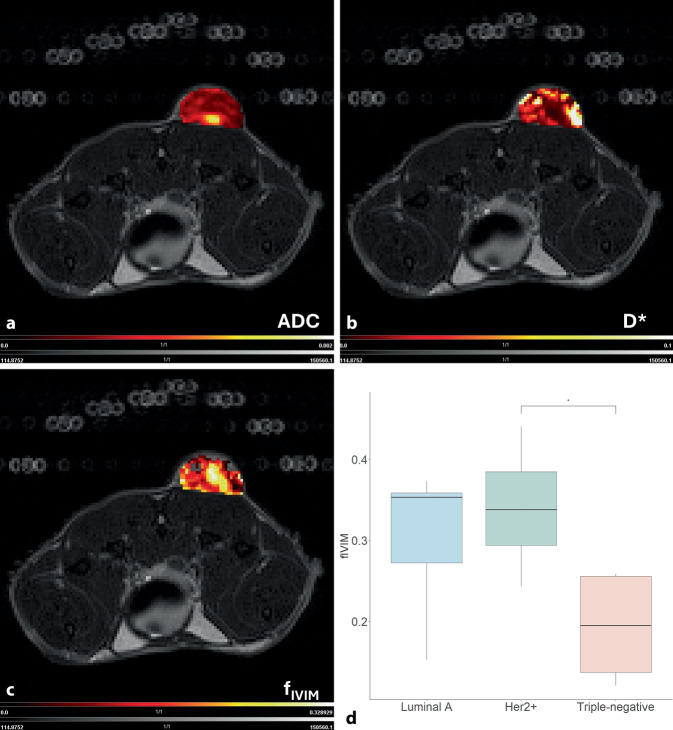
Übereinanderlagerung einer *T*_*2*_-gewichteten, axialen Ansicht des subkutanen HER2+ Brustkrebs-Xenografttumors im Mausmodell mit Parameterkarten des ADC (**a**), IVIM-Perfusionskoeffizienten D* (**b**) und des IVIM-Fraktionskoeffizienten f_IVIM_. **c** Der Vergleich der f_IVIM_ zwischen Brustkrebssubtypen im Boxplot zeigt die Unterschiede zwischen Luminal A, HER2+ und triple-negativen Brustkrebssubtypen (**d**). (Mod. nach [11], *p < 0,05)

Für b‑Werte unter 200 s/mm^2^ dominiert der Signalbeitrag der Perfusion in kleinen Blutgefäßen das Signal der molekularen Diffusion. Hierbei wird angenommen, dass sich der Blutfluss in den kleinen Gefäßen pseudozufällig verhält und dabei um das 10-fache schneller als die reine Diffusion ist. Um diesem Effekt Rechnung zu tragen kann ein biexponentielles Modell angewandt werden, das das monoexponentielle Modell zur Berechnung des ADC um die Parameter der Pseudodiffusion D* (Abb. 2b) und der Perfusionsfraktion f_IVIM_ (Abb. 2c) erweitert. Dieses Modell beschreibt den IVIM-Effekt und ermöglicht es, diffusive und perfusive Eigenschaften des Gewebes voneinander getrennt zu quantifizieren [15]. Das gängige DWI-Verfahren muss dazu lediglich um die zusätzliche Akquisition von einigen niedrigen b‑Werten ergänzt werden. Der Vorteil der IVIM-MRT liegt darin, dass kleine Blutgefäße, wie sie häufig in Tumoren vorkommen und die nicht durch herkömmliche DWI erfasst werden, auch ohne Gadolinium-basierte Kontrastmittel charakterisiert werden. Die DWI und somit auch die IVIM-MRT, sind jedoch für Feld- und Bewegungsartefakte relativ anfällig, welche durch die Verwendung spezialisierter Pulssequenzen teilweise eliminiert werden können [16].

Da die b‑Werte von Gradientenstärke, Gradientendauer und der Diffusionszeit (Abstand zwischen den beiden Gradienten) abhängen, können numerisch idente b‑Werte verschiedene Diffusionsgewichtungen haben und zu unterschiedlichen Signalen führen [17]. Dies erschwert deren Interpretation und Vergleichbarkeit vor allem im Rahmen multizentrischer Studien, die in aktuellen Untersuchungen thematisiert wird [18].

### Anwendung bei Brustkrebs

Die IVIM-Methode wurde zum ersten Mal im Jahr 2011 von Sigmund et al. [19] in der klinischen Brustdiagnostik angewandt. In der routinemäßigen klinischen Bildgebung hat sie jedoch bisher keine wesentliche Bedeutung. Präklinische Studien fokussieren sich auf die Modifizierung von der IVIM-MRT mit dem Ziel einer verbesserten Brustkarzinomcharakterisierung. Cho et al. [20] konnten zeigen, dass die IVIM-MRT mit dem Hormonrezeptorstatus und histologischen Markern von Brusttumoren korreliert. Ebenso konnte gezeigt werden, dass die Wahl der Diffusionszeit vor allem bei aggressiveren Tumortypen, wie dem triple-negativen Karzinom [17], einen großen Einfluss auf die IVIM-Parameter hat. Außerdem wurde IVIM-MRT in Kombination mit den oben beschriebenen BOLD-Parametern evaluiert. Dabei konnte gezeigt werden, dass eine Unterscheidung von molekularen Brustkrebssubtypen wie Luminal A, HER2+ und triple-negativen Karzinomen möglich ist (Abb. 2; [11]). So sinkt der IVIM-Fraktionskoeffizient (f_IVIM_) mit steigender Aggressivität des Tumortyps als Folge einer geringeren funktionellen Durchblutung des Tumorgewebes (Abb. 2d). Solche präklinischen Studien unterstreichen das hohe Potenzial der IVIM-MRT für die klinische Bildgebung. Eine konsequente Standardisierung des b‑Werte-Schemas und des Auswerteprotokolls ist jedoch notwendig, um die IVIM-MRT in der klinischen Anwendung erfolgreich zu etablieren [16].

## Von MRS zu Deuterium-MRT

### Magnetresonanzspektroskopie (MRS)

Die MRS ist ein bildgebendes Verfahren, mit dem die spezifischen Signale von Molekülen, in welche der ^1^H‑Kern eingebunden ist, erfasst werden können. Durch die Frequenzverschiebung des gemessenen Protonensignals kann man Rückschlüsse auf die chemische Zusammensetzung der Moleküle ziehen. Neben ^1^H werden auch alternative Kerne wie ^19^F, ^23^Na, ^31^P oder ^13^C für die MRS verwendet [1, 21]. In der Brustbildgebung wird meist der bereits bei 3 T sichtbare Marker Cholin (Cho) verwendet, der in aktiv proliferierenden Tumoren erhöht ist [22]. Bei dieser Feldstärke konnte ebenfalls zwischen benignen und malignen Läsionen differenziert werden [1]. Für alternative Kerne ist jedoch das MRS-Signal bei einer Feldstärke von 3 T zu niedrig, weswegen hier eine Feldstärke von 7 T benötigt wird [1, 23]. Zaric et al. [23] zeigten das Potenzial der ^23^Na-MRS mit der Differenzierung von benignen und malignen Brusttumoren in der 7‑T-MRT auf. Dennoch ist die breite Anwendung der MRS in der Klinik aufgrund von verhältnismäßig niedrigen verfügbaren Feldstärken, ihrer Sensibilität auf mögliche Feldinhomogenitäten und dem daraus resultierenden schwachen Signal der alternativen Kerne, limitiert. Die ^13^C‑MRS wurde zur hyperpolarisierten ^13^C‑MRS weiterentwickelt, welche durch die Hyperpolarisierung das gemessene Signal in der MRS erhöht. Limitiert ist diese Methode jedoch durch hohe Kosten und Komplexität der Herstellung von hyperpolarisierten Stoffen [21].

### Deuterium-MRT

**Abb. 3:**
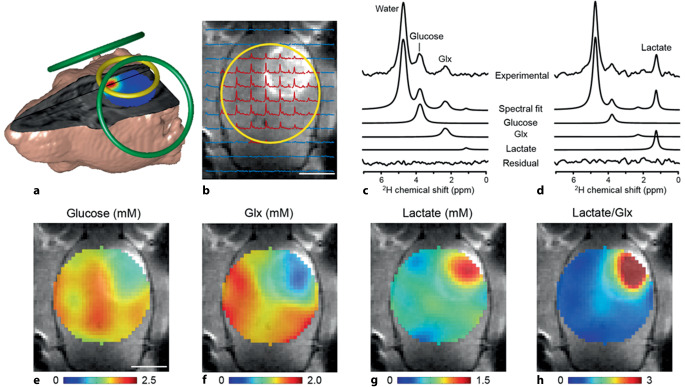
**a** Rattenmodell mit Oberflächenspulen für ^1^H‑MRT und Shim (*grün*) und Deuterium-MRT (DMI; *gelb*). **b** DCE‑T_1_-gewichtetes MRT-Bild mit hyperintensivem Gliom, Position der DMI-Spule mit *gelbem Ring* markiert und DMI-Spektren (2 × 2 × 2) mm^3^ Voxel. **c** DMI-Spektrum eines gesunden Voxels. **d** DMI-Spektrum im Gliom. **e**–**h** DMI-Maps mit Fits der Metaboliten Glukose, Glx (Glutamate und Glutamine), Laktase und der Laktase/Glx-Fraktion. (Aus [26], mit freundl. Genehmigung © AAAS)

Die DMI („deuterium metabolic imaging“) ist eine vielversprechende Methode zur Stoffwechselbildgebung. Basierend auf ersten Ansätzen aus den 1980er-Jahren rückte die DMI, begünstigt von immer höheren verfügbaren Magnetfeldstärken, in den letzten Jahren verstärkt in den Fokus der MRT-Forschung. Deuterium (^2^H) ist ein stabiles Wasserstoffisotop, das im Zuge der Deuterierung von Substanzen anstelle von Protonen (^1^H) gesetzt werden kann. Dabei ändern sich die chemischen Eigenschaften der deuterierten Substanzen nicht. DMI kann nicht nur Aufnahme und Transport der deuterierten Substanz verfolgen, sondern auch die dabei entstehenden deuterierten Metaboliten quantifizieren. Im resultierenden Spektrum kann man, ähnlich wie bei der MRS, das Signal der deuterierten Metaboliten durch Analyse dieses Spektrums voneinander unterscheiden (Abb. 3c, d). Mittels Baseline-Messungen vor der eigentlichen Untersuchung können außerdem natürlich vorhandene deuterierte Metaboliten (0,0115 % des Wasserstoffs liegt in Form von Deuterium vor) im zu untersuchenden Organ gemessen werden, was eine etwaige Verzerrung der Ergebnisse verhindert.

Deuterium ist aufgrund des vergleichsweise niedrigen gyromagnetischen Verhältnisses nicht anfällig für Signalschwankungen aufgrund von Magnetfeldinhomogenitäten [21]. Somit hat DMI eine relativ hohe Sensitivität sowie spektrale Auflösung und erfordert zudem keine Fett- oder Wasserunterdrückung. Im Gegensatz zu Positronen-Emissions-Tomographie (PET) und hyperpolarisierter ^13^C‑MRS ist die DMI eine technisch einfach umsetzbare Bildgebungsmethode, für die lediglich eine speziell abgestimmte Spule notwendig ist.

Die präklinische onkologische Forschung fokussiert sich derzeit auf die Erfassung des Glukosemetabolismus und der Laktaseproduktion [24, 25]. Dabei werden oral verabreichte deuterierte Stoffe wie ^2^H‑Glukose, ^2^H‑Cholin, ^2^H‑Fumarat, ^2^H‑Pyruvat und ^2^H‑3OMG (3-O-Methyl-D-Glukose) eingesetzt [25]. Eine richtungsweisende Studie aus dem Jahr 2018 konnte zum ersten Mal durch DMI erfolgreich den Glukosemetabolismus (Abb. 3c, e) und die dadurch resultierende Laktaseproduktion (Abb. 3d, g) in einem Gliom-Modell in der Ratte (Abb. 3) sowie im menschlichen Gehirn nachweisen [26].

### Anwendung bei Brustkrebs

DMI wurde neben Gliom- und Lymphom-Modellen auch in Brustkrebsmodellen angewandt [27]. In Brustkrebsmodellen konnte durch die steigende Malat/Fumarat-Fraktion die Schädigung und Apoptose der Zelle nach erfolgter Therapie dargestellt werden [28]. Die dynamische Bildgebung des Glukosestoffwechsels mit deuterierter 3‑OMG wurde in einem präklinischen metastasierenden Brustkrebsmodell bereits mit sehr hoher zeitlicher Auflösung (5s) in Ratten angewandt und könnte damit eine potenzielle Alternative zur [^18^F]-Fluordesoxyglukose-PET ([^18^F]FDG-PET) sein [29]. Die dynamische DMI wurde vor Kurzem von Niess et al. [30] im Gehirn angewandt und konnte regionale Unterschiede des Glukosemetabolismus darstellen. Somit scheint die Anwendung von deuterierten Glukosederivaten und Stoffwechselprodukten des Glukosemetabolismus auch in der Brustkrebsbildgebung vielversprechend.

## CEST-MRT

Die CEST-MRT („chemical exchange saturation transfer“) ist eine Methode für die Quantifizierung chemischer Austauschprozesse im Gewebe und beruht auf dem Transfer von Magnetisierung zwischen einem Austauschmolekül und dem umgebenden Wasser (H_2_O).

Dazu werden Protonen eines Moleküls mit einem Radiofrequenzpuls saturiert, während ein chemischer Austausch der Protonen mit jenen des umgebenden Wassers stattfindet und somit auch dessen Signal reduziert [31].

Ein Vorteil der CEST-MRT im Gegensatz zur MRS ist die höhere örtliche Auflösung, da hier schon geringe Konzentrationen des Zielmoleküls zu einem messbaren Signal führen. Außerdem benötigt man bei CEST-MRT im Gegensatz zu DMI keine zusätzlichen technischen Voraussetzungen wie speziell abgestimmte Spulen. Ein Signal geben jedoch nur jene Moleküle, die Protonen für den chemischen Protonenaustausch mit Wasser besitzen und in denen die Resonanzfrequenz der austauschbaren Protonen einen ausreichenden Abstand zur Resonanzfrequenz der Wasserprotonen aufweisen. Aus der großen Vielfalt der CEST-basierten Methoden kommen vor allem glucoCEST, APTw MRT und acidoCEST für die Brustkrebsbildgebung in Frage, welche im Folgenden näher beschrieben werden sollen.

### glucoCEST-MRT

Die Quantifizierung des Glukosemetabolismus spielt in der Krebsforschung eine wichtige Rolle und hat die Entwicklung der [^18^F]FDG-PET motiviert. Eine MRT-basierte Methode zur Quantifizierung des Glukosemetabolismus ist die glucoCEST-MRT. Dabei werden nicht radioaktiv markierte Glukosederivate injiziert, die mittels CEST-MRT im Tumorgewebe nachgewiesen werden können. Eines der vielversprechendsten Derivate ist die 2‑Desoxy-D-Glukose (2-DG), die dem alltäglich eingesetzten [^18^F]FDG ähnlich ist. Durch den Protonenaustausch an die glukosegebundenen Hydroxylgruppen mit dem umgebenden Wasser wird ein lokalisierbares Glukosesignal gemessen [32].

### APTw MRT

Die Amide-Protonen-Transfer-gewichtete (APTw) MRT basiert auf dem CEST-Signal von sich in Proteinen befindlichen Amiden [33]. Aufgrund der endogenen Häufigkeit von Amiden und der pH-Abhängigkeit des Protonenaustauschs, wurde diese kontrastmittelfreie Methode zur MRT-basierten Bildgebung von pH-Veränderungen nach einem ischämischen Schlaganfall im Rattenhirn erstmals vorgestellt [33]. Auch in Brusttumoren könnte diese nichtinvasive Methode den pH-Wert, der ein bekannter prognostischer Faktor ist, bestimmen [34].

### acidoCEST-MRT

Viele bildgebende Methoden, die den pH-Wert *in vivo *darstellen, wurden bereits untersucht, wobei alle mit aufwändigen Messprotokollen und anderen nennenswerten Schwierigkeiten einhergehen [34]. Neben der oben beschriebenen APTw-MRT ist die acidoCEST-MRT eine der vielversprechendsten Methoden. Hierbei werden exogene Kontrastmittel, die einen pH-abhängigen CEST-Kontrast generieren, verabreicht. Iopamidol, ein für die klinische Diagnostik zugelassenes CT-Kontrastmittel, wurde 2011 erstmals präklinisch *in vivo* getestet [35].

### Anwendung bei Brustkrebs

**Abb. 4:**
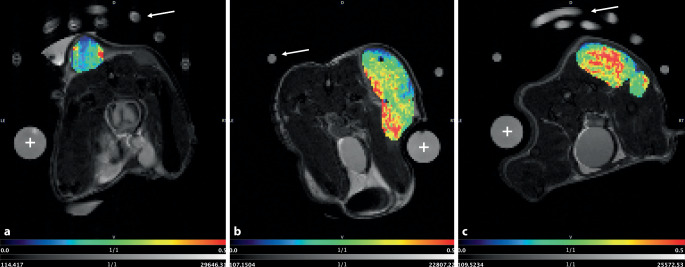
2‑DG glucoCEST-MRT „apparent exchange-dependent relaxation“ (AREX) Parameterkarten (überlagert auf ein *T*_*2*_-gewichtetes, axiales anatomisches Referenzbild) für jeweils einen Luminal A (**a**), HER2+ (**b**) und triple-negativen (**c**) subkutanen Brustkrebs-Xenografttumor im Mausmodell mit Glukosephantom (+) und dorsal positioniertem Wärmebett (*Pfeil*). *Blaue* Bereiche beschreiben Areale mit niedrigem Glukosestoffwechsel (dominant im wenig aggressiven Luminal-A-Tumor). *Rote* Areale beschreiben metabolisch sehr aktive Areale, die vermehrt in den aggressiveren triple-negativen Karzinomen vorkommen. Im Gegensatz dazu dominieren als Folge einer mittelgradigen Aggressivität des Tumortyps HER2+, die Farben *Gelb* und *Hellgrün*

Der Glukosemetabolismus ist in der Brustkrebsdiagnostik ein wichtiger Biomarker, der mittels glucoCEST-MRT quantifiziert werden kann. Durch erweiterte fit-basierte sowie T_1_-korrigierte („AREX“; Abb. 4) Auswertemethoden [36] können Spezifität und Sensitivität für zuvor in der Präklinik identifizierte Glukoseaustauschpools erhöht und somit die Brustkrebsbildgebung verbessert werden. Mithilfe dieser fortgeschrittenen Auswertungsmethode wird die Heterogenität des Tumors anhand der glucoCEST-Parameterkarten (Abb. 4) sichtbar. Ob diese Methode darüber hinaus eine Differenzierung der molekularen Brustkrebssubtypen zulässt, ist Gegenstand aktueller präklinischer Forschung.

Die APTw-MRT wurde in der Brustkrebsbildgebung bisher nur experimentell angewandt [37]. Hierbei zeigte sich, dass die Signalinterferenz des umliegenden Fettgewebes eine sinnvolle Quantifizierung des Protonenaustauschsignals erschwert [38]. In den vergangenen Jahren sind Fortschritte in der Fettkorrektur erreicht worden, die eine zukünftige Anwendung von APTw-MRT in der Brustbildgebung ermöglichen werden [39]. Durch APTw-MRT kann die Heterogenität des pH-abhängigen Amidesignals in präklinischen Brustkrebsmodellen jedoch schon jetzt sichtbar gemacht werden.

Auch die pH-sensitive acidoCEST-Methode scheint vielversprechend für die Brustkrebsbildgebung zu sein, da vor allem in Hinsicht auf die Erfassung des Therapieansprechens der pH-Wert einer der ersten Biomarker ist, der eine Änderung zeigt. Die acidoCEST-MRT wurde im Jahr 2017 zum ersten Mal in der klinischen Brustbildgebung angewandt [40].

Es ist festzuhalten, dass für all diese Methoden ein standardisiertes und vor allem schnelles Mess- und Auswerteprotokoll etabliert werden muss.

## Ausblick

Die mpMRT hat bereits einen zentralen Stellenwert in der Brustkrebsdiagnostik, und die gezeigten präklinischen Ergebnisse von neuen bzw. modifizierten MRT-Methoden wie BOLD-MRT, IVIM-MRT, DMI und CEST-MRT werden durch die Anwendung in der klinischen Brustbildgebung laufend erweitert. Mit diesen Methoden ist bereits jetzt präklinisch eine nichtinvasive Differenzierung von molekularen Brustkrebssubtypen sowie die wiederholte Erfassung der intratumoralen Heterogenität während des Therapieverlaufs möglich. Eine effiziente Kombination der daraus resultierenden Parameter in mpMRT-Protokollen wird daher zunehmend eine klinische Brustbildgebung ohne die Anwendung Gadolinium-haltiger Kontrastmittel ermöglichen. Weitere systematische Evaluierungen der Quantifizierungsmethoden und Standardisierung der MRT-Parameter sind aber noch notwendig. Am Ende gilt es, mit den in der Präklinik gewonnenen Erkenntnissen die Translation zur klinischen Anwendung zu erreichen. Das Ziel ist es, durch die Etablierung eines standardisierten und auf mpMRT basierenden Bildgebungsregimes die nichtinvasive Brustkrebsdiagnostik zu verbessern und zu personalisieren.

## Fazit für die Praxis

- Die multiparametrische Magnetresonanztomographie (mpMRT) ermöglicht die nichtinvasive bildgebende Charakterisierung verschiedener Schlüsselprozesse der Krebsentstehung wie Hypoxie, Neoangiogenese und metabolische Veränderungen.
- Die ersten präklinischen Ergebnisse zu neuen bzw. optimierten MRT-Methoden wie BOLD („blood oxygen level dependent“), IVIM („intravoxel incoherent motion“), DMI (Deuterium-MRT) und CEST („chemical exchange saturation transfer“) sind vielversprechend.
- Die mpMRT, vor allem als Kombination von funktionellen, metabolischen und molekularen bildgebenden Methoden wird in Zukunft eine zentrale Rolle in der klinischen Brustdiagnostik einnehmen.
- Die präklinische Forschung und eine erfolgreiche Translation der damit gewonnenen Erkenntnisse bilden somit die Grundlage neuer mpMRT-Protokolle in der Klinik.
